# Outcomes in Adenoid Cystic Carcinoma: A 14-Year Analysis

**DOI:** 10.7759/cureus.75222

**Published:** 2024-12-06

**Authors:** Abdulrahman Bin Sumaida, Nandan M Shanbhag, Khalifa AlKaabi, Syed Mansoor Hasnain, Khalid Balaraj

**Affiliations:** 1 Oncology: Radiation Oncology, Tawam Hospital, Al Ain, ARE; 2 Internal Medicine, College of Medicine and Health Sciences, United Arab Emirates University, Al Ain, ARE; 3 Radiation Oncology: Palliative Care, Tawam Hospital, Al Ain, ARE; 4 Radiation Oncology, Tawam Hospital, Al Ain, ARE

**Keywords:** adenoid cystic carcinoma, disease-free survival, oncology, overall survival, prognostic factors, recurrence patterns, retrospective study, salivary gland tumors, survival analysis, treatment modalities

## Abstract

Background

Adenoid cystic carcinoma (ACC) is a rare malignancy characterized by slow progression, local recurrence, and distant metastases. This study aims to evaluate the demographic patterns, clinical presentations, outcomes, and survival trends of patients with ACC.

Methods

A retrospective analysis of 14 patients diagnosed with ACC from 2010 to 2024 at a tertiary cancer center in the United Arab Emirates was conducted. Data on demographics, presenting symptoms, clinical outcomes, tumor characteristics, recurrence patterns, and survival were analyzed. Kaplan-Meier survival analysis was performed to evaluate survival trends.

Results

The cohort was predominantly male (64.3%, n=9) with a mean age of 38.9 years. An enlarging mass in the neck (50%, n=7) was the most common presenting symptom. Local recurrence and distant metastases were observed in 42.9% (n=6) of patients each, with the lungs being the most frequent site of metastases (83.3%, n=5). Advanced-stage disease (Stage III/IV) was common (64.3%, n=9). Overall survival was 85.7% (n=12) over a mean follow-up of 4.3 years. Smoking was associated with poorer survival trends, though not statistically significant (HR=7.0, p=0.083).

Conclusion

ACC predominantly affects middle-aged individuals and is associated with high rates of local recurrence and distant metastases, especially to the lungs. Although survival trends varied by gender, smoking, and metastases, these differences were not statistically significant, underscoring the need for larger studies to better elucidate prognostic factors.

## Introduction

Adenoid cystic carcinoma (ACC) is a rare malignant tumor that primarily originates from the salivary glands, accounting for approximately 1% of all head and neck malignancies and 10% of salivary gland tumors [1]. Despite its relatively low incidence, ACC is characterized by a distinct clinical and pathological profile, including perineural invasion, slow progression, and a high propensity for distant metastases, which makes it a challenging disease to manage [2]. Long-term follow-up data highlight the aggressive nature of ACC, with significant morbidity and mortality resulting from recurrent and metastatic disease, even after initial treatment [3].

The clinical management of ACC involves a multidisciplinary approach, typically combining surgery with adjuvant radiotherapy to achieve local control. However, due to the tumor’s resistance to conventional systemic therapies and the frequent occurrence of late recurrences, the prognosis for patients remains guarded [4]. Understanding the factors that influence outcomes, such as overall survival (OS) and disease-free survival (DFS), is critical for optimizing treatment strategies and improving patient care. Previous studies have highlighted the prognostic value of clinical and demographic parameters, including age at diagnosis, performance status, and tumor stage, as well as the impact of treatment modalities on survival outcomes [5]. Nevertheless, there remains a paucity of comprehensive data on ACC, particularly from large tertiary care oncology centers that provide long-term follow-up.

This retrospective observational study was conducted at a high-volume oncology center to address this gap by analyzing the clinical, demographic, treatment, and outcome data of patients with ACC over a 14-year period. The study aims to identify factors associated with survival and recurrence patterns and evaluate the effectiveness of various treatment modalities in improving outcomes. By leveraging a robust institutional database, this study can provide valuable insights into the natural history and management of ACC in a real-world setting. In addition to advancing the understanding of ACC, the findings of this study have practical implications for improving prognostication and personalizing treatment strategies. The research discusses the recurrence pattern in ACC and the following definitions are implied. Local recurrence refers to the re-emergence of malignancy at or within the primary tumor site after definitive treatment, indicating residual microscopic disease or incomplete local control. Regional recurrence involves the recurrence of malignancy in the lymph nodes or adjacent structures within the drainage basin. Distant metastases denotes the dissemination of malignancy to distant organs or structures. In ACC, the lungs are the most common site of distant metastasis, with less frequent involvement of the bones, liver, or brain. This study also adheres to the highest ethical standards, ensuring patient confidentiality and compliance with international guidelines for retrospective research.

## Materials and methods

Study design

This was a retrospective observational study analyzing the clinical, demographic, treatment, and outcome data of patients diagnosed with ACC over 14 years, from August 2010 to October 2024. It was conducted at Tawam Hospital, Al Ain, United Arab Emirates, a tertiary care oncology center, and all data were extracted from the institutional database.

Study population

The dataset included anonymized records of patients diagnosed with ACC. Inclusion criteria for this study were a confirmed diagnosis of ACC based on histopathological findings and patients treated and followed up at the tertiary care center during the study period. Exclusion criteria included incomplete records and patients diagnosed with other histological subtypes of salivary gland tumors.

Data collection

Demographics: age at diagnosis, gender, smoking history, and alcohol consumption. Clinical presentation: performance status (Eastern Cooperative Oncology Group or ECOG scale), and presenting symptoms such as pain, hearing loss, or facial nerve palsy. Diagnostic data: date of diagnosis, imaging, and pathological reports. Treatment details: surgical interventions, radiation therapy, systemic treatments, and salvage procedures for recurrence or metastases. Outcome data: local, regional, and distant recurrences, as well as survival status at the last follow-up.

Outcomes

Primary outcomes: OS, DFS, and recurrence patterns (local, regional, and distant). Secondary outcomes: Treatment modalities and their relationship to outcomes.

Statistical analysis

Descriptive statistics were used to summarize demographic and clinical characteristics. Kaplan-Meier survival curves were generated to evaluate OS and DFS. Categorical variables, such as recurrence patterns, were analyzed using chi-square or Fisher’s exact tests. Associations between clinical parameters (e.g., ECOG score, treatment modality) and outcomes were evaluated using Cox proportional hazards models. A p-value <0.05 was considered statistically significant.

Ethical considerations

The study was approved by the Tawam Human Ethics and Research Committee and assigned approval number MF2058-2024-181. All patient data were anonymized to ensure confidentiality and privacy. Data were securely stored and analyzed in compliance with institutional and international ethical guidelines for retrospective studies.

## Results

Demographics and presenting symptoms

The study cohort consisted of 14 patients diagnosed with ACC, with a male predominance (64.3%, n=9) compared to female patients (35.7%, n=5). The mean age at diagnosis was 38.9 years (range 22-60 years), with the majority of patients in the 30-50 year age group. Among the lifestyle factors assessed, 42.9% of patients reported they were smokers while none consumed alcohol. Presenting symptoms included mass in the neck (50%, n=7) and pain (21.4%, n=3), with hearing loss and facial nerve palsy notably absent in the cohort. These findings are summarized in Table 1, which highlights the demographic characteristics and distribution of presenting symptoms.

**Table 1:** Demographic and clinical characteristics of the study cohort This table summarizes the demographic profile and initial presenting symptoms of patients diagnosed with adenoid cystic carcinoma. Total number of patients=14. *Age at diagnosis is mentioned in years.

Demographic or symptom	Number of patients (%)*
Male	9 (64%)
Female	5 (26%)
Smokers	6 (43%)
Non-smokers	8 (57%)
Mean age at diagnosis (years)	38.9 yrs
Mass in the neck as the presenting symptom	7 (50%)
Pain as the presenting symptom	3 (21%)
Hearing loss as the presenting symptom	0
Facial nerve palsy as the presenting symptom	0

Clinical outcomes

Among the study cohort, regional recurrences were observed in 14.3% (n=2) of patients, all of whom received appropriate treatment (surgery, radiotherapy, and combination treatment). Local recurrence was present in 42.9% (n=6) of patients, with the remaining 57.1% (n=8) being recurrence-free. None of the patients experienced any regional recurrence during the follow-up period. Distant metastases were present in 42.9% (n=6) of patients, with the remaining 57.1% (n=8) being metastasis-free. At the time of the last follow-up, 85.7% (n=12) of patients were alive, while 14.3% (n=2) had succumbed to their illness. The mean follow-up duration was 4.3 years (range 0.24-8.65 years) (Table 2).

**Table 2:** Clinical outcomes of the study cohort Total number of patients=14. *Mean follow-up duration given in years.

Clinical outcome	Number (%)
No local recurrence	8 (57%)
Local recurrence which was treated	6 (43%)
Presence of distant metastases	6 (43%)
Absence of distant metastases	8 (57%)
Alive at the last follow-up	12 (86%)
Deceased at the last follow-up	2 (14%)
Mean follow-up duration (years)	4.3 yrs

Primary site

The submandibular gland was the most common site, accounting for 50% (n=7) of cases. This was followed by the parotid gland and the floor of the mouth, each comprising 14.3% (n=2) of cases. The less frequent tumor sites included the maxilla (7.1%, n=1), mandible (7.1%, n=1), and the trachea (7.1%, n=1).

Stage analysis and recurrence pattern

The cohort demonstrated a varied distribution across clinical stages at diagnosis. The majority of patients were diagnosed at advanced stages, with Stage III accounting for 42.9% (n=6) and Stage IV comprising 21.4% (n=3). Early-stage presentations were less common, with Stage I observed in 21.4% (n=3) of patients. The stage at diagnosis was unknown for 14.3% (n=2) of the cohort. 

Local recurrence occurred in six patients (42.9%). Treatments for local recurrence varied, with salvage surgery being the most common treatment (33.3%, n=2), followed by salvage surgery and then re-irradiation (16.7%, n=1), palliative radiotherapy (16.7%, n=1), re-irradiation alone (16.7%, n=1), and rigid bronchoscopy with debulking (16.7%, n=1).

Six patients (42.9%) had distant metastases, with the most common metastatic sites being the lungs (83.3%, n=5) and the brain (16.7%, n=1). Regional recurrence was not observed in any patient during the study period. However, distant metastases occurred during follow-up in six patients (42.9%). Among these patients, treatments included palliative radiotherapy (50%, n=3), palliative chemotherapy (16.7%, n=1), and salvage surgery of oligometastases (16.7%, n=1).

Time to metastasis from the date of diagnosis

Among patients who developed distant metastases, the median time to distant failure from diagnosis was 12.2 months, with a mean of 31.7 months. The timing ranged from zero months (indicating that metastases were already present at diagnosis) to 80.7 months after diagnosis. The standard deviation was 36.3 months.

Survival analysis**

Survival by Gender

**Figure 1:**
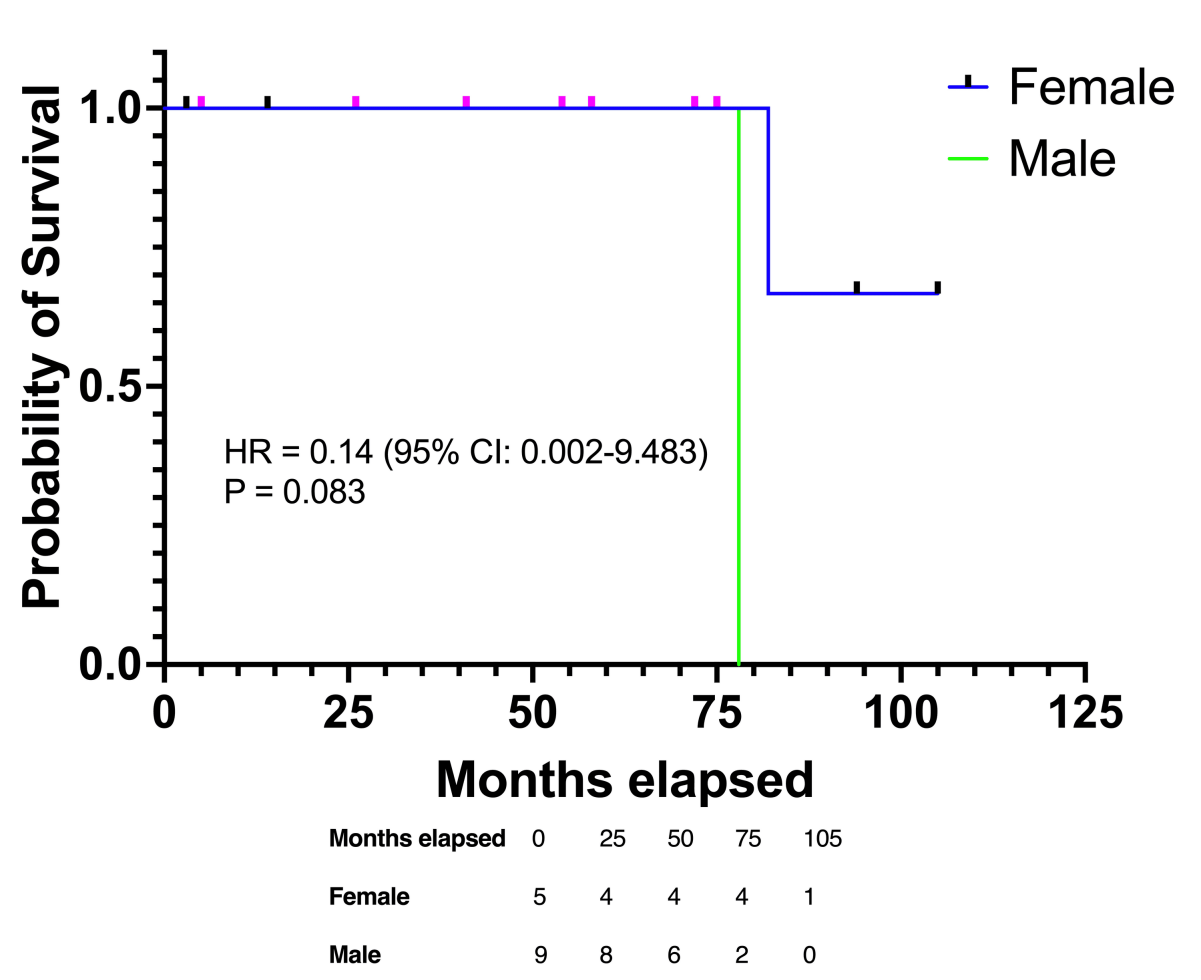
Kaplan-Meier survival curve based on the gender of the patients The figure compares the survival of males and females in the cohort. Significance level p<0.05

The Kaplan-Meier analysis stratified by gender demonstrated differences in survival trends between the male and female patients. At the time of diagnosis, there were nine male and five female patients. Over the follow-up period, the survival probabilities for males declined more steeply in the earlier years compared to females. By eight years, both groups converged, with one patient from each gender remaining at risk. The hazard ratio (HR) for female patients compared to males was 0.14 (95% CI 0.002-9.483, p=0.083), indicating no statistically significant difference in survival based on gender (Figure 1).

Survival by Distant Metastases

**Figure 2:**
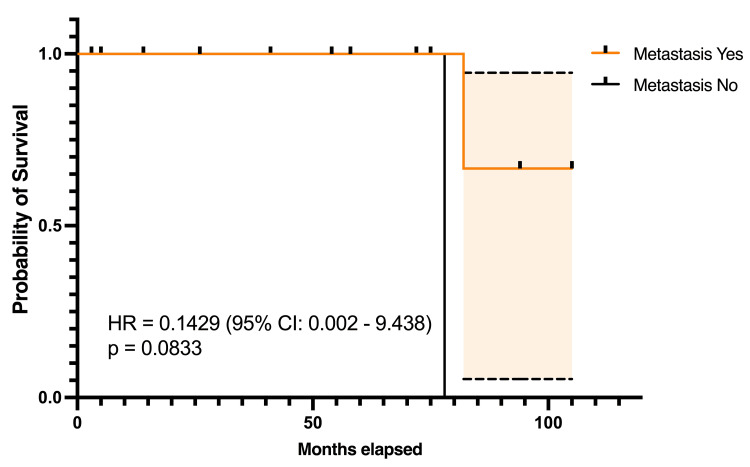
Kaplan-Meier survival curve for patients with or without metastases Significance level p<0.05.

In the survival analysis comparing patients with and without metastases, the log-rank test did not reveal a statistically significant difference in survival curves (p=0.0833). The HR was 0.1429 (95% Cl 0.002 9.438)(Figure 2).

Survival by Smoking Status

**Figure 3:**
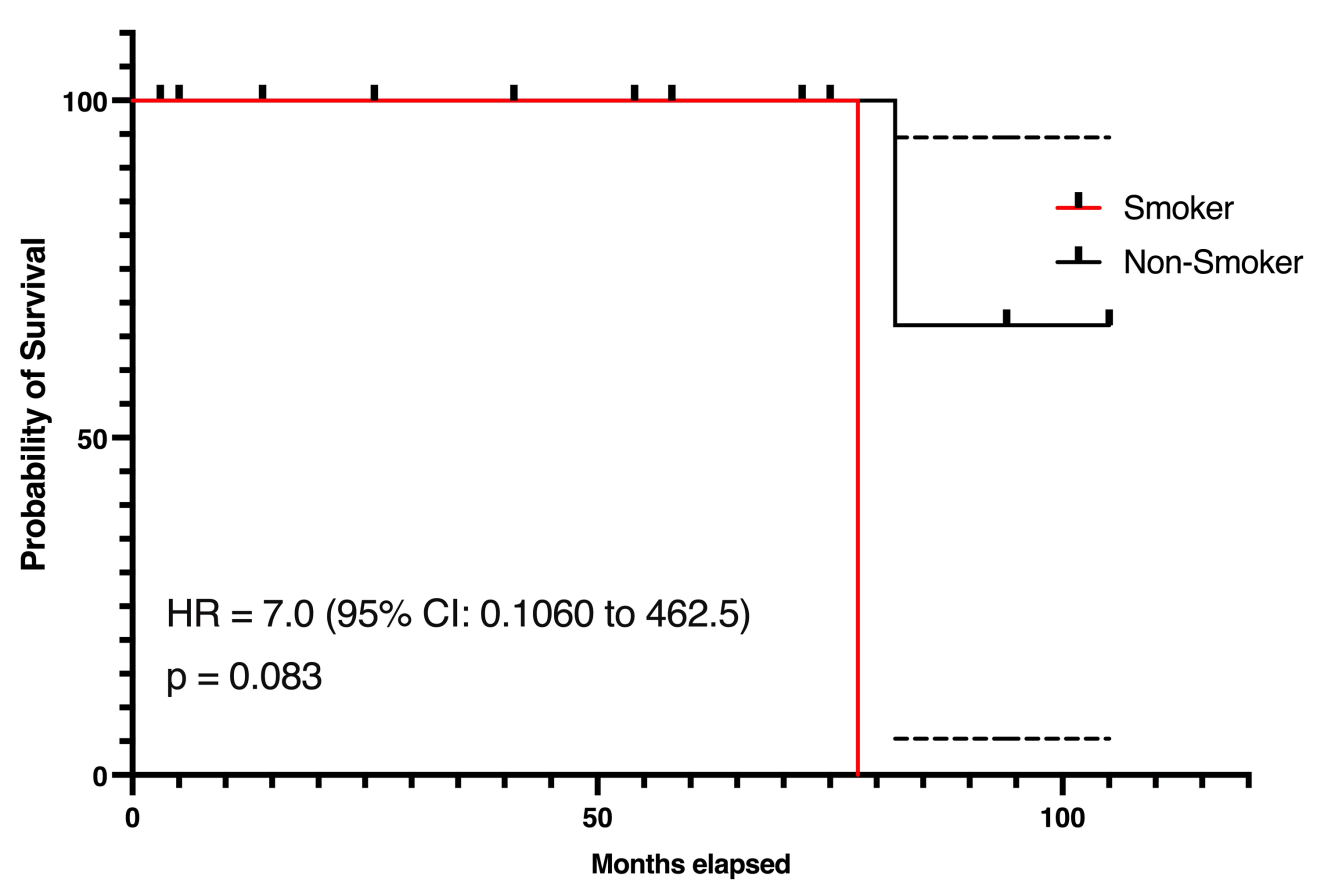
Kaplan-Meier survival curves for smokers and non-smokers in the study cohort Significance level at p<0.05

The survival analysis stratified by smoking status revealed differences in survival outcomes. Non-smokers exhibited a more favorable survival probability compared to smokers throughout the follow-up period. By six years, the number of smokers at risk had decreased significantly, with only one smoker remaining at eight years. Non-smokers showed a more gradual decline in survival. The hazard ratio for smokers compared to non-smokers was seven (95% CI 0.106-462.5, p= 0.083), indicating that smokers had a higher risk of death compared to non-smokers. However, this trend toward reduced survival was not statistically significant (Figure 3).

## Discussion

This study provides a detailed analysis of the demographics, clinical presentation, outcomes, and survival trends in patients diagnosed with ACC. The findings provide insights into the disease’s behavior and highlight several trends related to recurrence, metastasis, and survival.

Demographics and presenting symptoms

The cohort demonstrated a male predominance with nine male (64.3%) compared to five female (35.7%) patients, consistent with other reports on ACC demographics [6]. The mean age at diagnosis was 38.9 years, with most patients (n=9, 64.3%) presenting in the 30 to 50-year age group, which aligns with the literature suggesting ACC affects middle-aged adults [7]. Smoking was reported in six patients (42.9%), suggesting a possible association between lifestyle factors and disease development, though none of the patients consumed alcohol. Notably, a mass in the neck was the most common presenting symptom, affecting seven patients (50%), while hearing loss and facial nerve palsy were not observed in any of them. This symptomatology is in line with the indolent yet locally aggressive nature of ACC [8].

Tumor characteristics and primary site

The submandibular gland was the most common primary site, accounting for seven cases (50%), consistent with the established patterns of salivary gland ACC [8]. Advanced stage presentations (Stage III and IV) were predominant in this cohort, with Stage III in six patients (42.9%) and Stage IV in three patients (21.4%). Early-stage presentations were less common, with Stage I observed in three patients (21.4%), while the stage at diagnosis was unknown for two patients (14.3%) [8].

Clinical outcomes

The clinical outcomes of the cohort highlight the disease's propensity for local and distant recurrences. Local recurrence was observed in six patients (42.9%), highlighting the challenges in achieving complete local control despite multimodal treatments [9]. The frequency of distant metastases, seen in six patients (42.9%), predominantly to the lungs (five patients, 83.3% of those with metastases), mirrors the findings from prior studies, emphasizing the metastatic proclivity of ACC, especially in advanced stages [10]. The OS rate of 85.7% (12 patients) at the last follow-up reflects the slow-growing but persistent nature of the disease.

Recurrence and metastases patterns

Local recurrences were managed with various modalities, including salvage surgery in two patients (33.3% of those with tumor recurrence), salvage surgery followed by re-irradiation, palliative radiotherapy, re-irradiation alone, and rigid bronchoscopy with debulking in one patient each (16.7%). The range of treatment strategies highlights the individualized nature of recurrence management in ACC [11].

Distant metastases occurred in six patients (42.9%), predominantly to the lungs (five patients, 83.3% of those with metastases) and the brain (one patient, 16.7%). Treatments for metastases varied, with palliative radiotherapy being the most commonly used approach (three patients, 50% of those with metastases), followed by palliative chemotherapy, and salvage surgery of oligometastases in one patient each (16.7%) [12].

The median time to distant failure was 12.2 months, with a mean of 31.7 months and a range of 0-80.7 months. Notably, metastases were already present at diagnosis in some cases, further highlighting the aggressive nature of certain ACC presentations [13].

Survival analysis

While survival probabilities differed based on gender, smoking status, and presence of metastases, none of these factors reached statistical significance. The HR for females suggested a trend toward better survival compared to males, though the difference was not statistically significant (HR 0.14, 95% CI 0.002-9.483, p=0.083) [6]. Smokers exhibited a poorer survival trend compared to non-smokers, with a HR of 7.0 (95% CI 0.106-462.5, p=0.083), emphasizing the potential role of smoking as a negative prognostic factor in ACC [7]. Patients without metastases demonstrated better survival probabilities, though the differences were not statistically significant, likely due to the small cohort size (HR 0.1429, 95% CI 0.002-9.438, p=0.0833) [9].

Management

Effective management requires a multimodal approach integrating surgery, radiotherapy, and in some cases, systemic therapies.

Surgical Management

Surgery remains the cornerstone of ACC treatment, especially for localized disease. Complete surgical resection with negative margins is crucial for long-term local control. However, achieving negative margins can be challenging due to the tumor's infiltrative growth and perineural spread. Radical parotidectomy or cranial base surgery may be necessary for advanced cases, but these procedures often involve risks such as facial nerve damage, which can be mitigated with reconstructive techniques [14].

Radiotherapy

Adjuvant radiotherapy is recommended for most patients, particularly those with positive surgical margins or perineural invasion. Studies have demonstrated improved local control rates with doses exceeding 60 Gy, particularly for cases with microscopic residual disease [15]. Intensity-modulated radiotherapy (IMRT) and proton therapy are emerging as effective modalities, offering precision targeting with reduced toxicity [16].

Chemotherapy and Targeted Therapies

Systemic chemotherapy plays a limited role in ACC due to its indolent nature and poor response to cytotoxic agents. However, palliative chemotherapy with combinations like cisplatin and 5-fluorouracil has shown some benefit in symptom control for advanced cases [17]. Targeted therapies, including tyrosine kinase inhibitors (e.g., vascular endothelial growth factor receptors or VEGFR inhibitors), immune checkpoint inhibitors, and novel agents targeting Notch signaling, are being investigated for metastatic disease [18]. Tyrosine kinase inhibitors (TKIs) targeting VEGFRs and other pathways have shown potential in slowing tumor progression in ACC. Lenvatinib, a multi-kinase inhibitor targeting VEGFR1-3, fibroblast growth factor receptor (FGFR) 1-4, and platelet-derived growth factor receptor (PDGFR), improved progression-free survival (PFS) in a phase II trial, achieving a median PFS of 9.1 months and an 88% disease control rate [19]. Similarly, axitinib, another VEGFR inhibitor, demonstrated disease stabilization in a subset of ACC patients [20]. Immune checkpoint inhibitors (ICIs), such as pembrolizumab, are also being explored in ACC due to their low mutational burden and programmed death-ligand 1 (PD-L1) expression. While pembrolizumab demonstrated limited efficacy in a phase II trial with an overall response rate of 12%, it provided disease stabilization in some patients, and ongoing investigations aim to enhance immune responsiveness through combination strategies [21]. In addition, the Notch pathway inhibitors targeting aberrant Notch signaling implicated in ACC pathogenesis have shown promise. AL101, a gamma-secretase inhibitor, demonstrated potential for disease stabilization in Notch-activated ACC patients, as indicated by preliminary data from the ACCURACY trial [22]. Histone deacetylase (HDAC) inhibitors, such as vorinostat, are also under investigation, with limited efficacy as monotherapies but show potential when combined with other agents [23]. 

Management of Metastatic and Recurrent Disease

Distant metastases, often to the lungs, are common in ACC and can occur years after initial treatment. Surgical resection of oligometastatic lung disease or stereotactic body radiation therapy (SBRT) may improve outcomes in select patients [24]. For unresectable metastatic disease, systemic therapies are palliative but offer limited efficacy.

Limitations

The small sample size (n=14) is a primary limitation of this study, affecting the statistical power and generalizability of findings. Additionally, the retrospective nature of the study introduces potential biases, including incomplete data on treatments and outcomes.

## Conclusions

This study highlights the clinical complexity of ACC, emphasizing its tendency for late-stage presentation, high recurrence rates, and distant metastases. Although survival trends varied by gender, smoking status, and metastases, these differences were not statistically significant, underscoring the need for larger, multicenter studies to elucidate prognostic factors. The findings reiterate the importance of early detection and tailored therapeutic approaches in managing ACC.
